# Treatment and complications of PCOS in adolescents - what’s new in 2023?

**DOI:** 10.3389/fendo.2024.1436952

**Published:** 2024-10-02

**Authors:** Karolina Jakubowska-Kowal, Karolina Skrzyńska, Aneta Gawlik-Starzyk

**Affiliations:** Department of Pediatrics and Pediatric Endocrinology, Medical University of Silesia, Katowice, Poland

**Keywords:** polycystic ovary syndrome, adolescent, oligomenorrhea, hirsutism, hyperandrogenism

## Abstract

Polycystic ovary syndrome (PCOS) is a disease affecting as many as about 10% of women of reproductive age, also 3-11% of teenage patients, and can lead to numerous complications and coexists with many diseases. Research is ongoing to establish an appropriate diagnostic and therapeutic path for adolescent girls with PCOS. It is also important to implement appropriate check-ups among teenagers with PCOS in order to prevent PCOS complications and initiate appropriate treatment as soon as possible and prevent the long-term consequences of these complications. The relationship between the co-occurrence of PCOS and diseases such as metabolic syndrome, hypertension, obesity, insulin resistance, type 2 diabetes and non-alcoholic fatty liver disease (NAFLD) is increasingly being investigated. A great attention is also being paid to the problem of mental health in this group of patients. In our study, we will review the latest reports on the treatment of PCOS and look at the complications that this syndrome can cause.

## 1 Introduction and background

Polycystic ovary syndrome (PCOS) is a disease affecting as many as about 10% of women of reproductive age, also 3-11% of teenage patients (1, 2). The etiology is multifactorial and is still an object of research by scientists. Genetic and environmental factors, disturbances in steroidogenesis, metabolic changes or neuroendocrine alterations may be responsible for the development of this syndrome (3). Also important is the increased concentration of follistatin, which is associated not only with PCOS, but also with other diseases that may co-occur with PCOS, such as type II diabetes (4). To diagnose PCOS in adult patients, it is necessary to meet two of the three Rotterdam criteria (oligoovulation or anovulation, clinical and/or biochemical hyperandrogenism, and polycystic ovarian ultrasonography) (5). Due to physiological differences among adolescent patients, we do not use the same criteria as in adult patients, because it may lead to overdiagnosis of this syndrome. Currently, it seems to be the most appropriate to use the criteria proposed by Ibanez L et al. according to which PCOS can be diagnosed when a teenage patient presents irregular menstrual cycles and biochemical and/or clinical hyperandrogenism. Menstrual disorders should be assessed 2 years after menarche and may manifest as oligomenorrhea (menstrual cycles longer than 45 days), dysfunctional uterine bleeding (cycles shorter than 21 days or lasting more than 7 days), and also as secondary amenorrhea (absence of cycles for more than 90 days), or primary amenorrhea. Biochemical hyperandrogenism is diagnosed if total testosterone concentrations >55 ng/dL (1.91 nmol/L), while clinical hyperandrogenism is diagnosed using the modified Ferriman-Gallwey scoring system (modified Ferriman–Gallwey mFG score ≥4–6) (3, 6) PCOS can lead to numerous complications and coexists with many diseases. Research shows that teenage PCOS patients can often concerning such health problems as metabolic syndrome, hypertension, obesity, insulin resistance, type 2 diabetes and non-alcoholic fatty liver disease (NAFLD; MAFLD metabolic-associated fatty liver disease is now a more commonly used name) (7–10). An important issue is also increased risk of depressive disorders and anxiety among young women and teenagers with PCOS (11, 12). In our study, we will review the latest reports on the treatment of PCOS and look at the complications that this syndrome can cause.

## 2 Methodology

**Figure 1:**
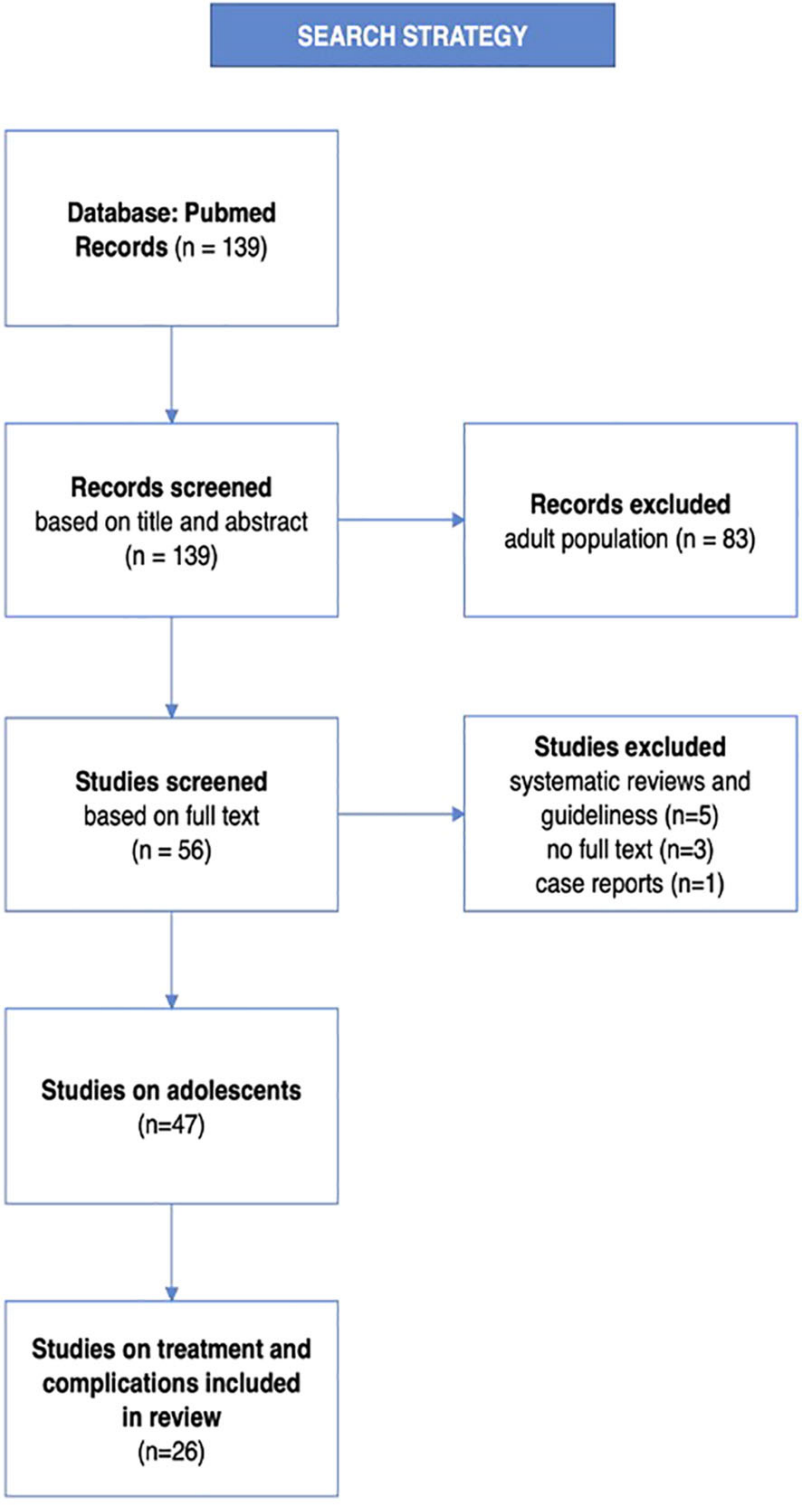
Search strategy presented on the flow diagram.

For this review, articles published in the PubMed database from 2022/07/01 to 2023/06/30 were searched using the terms (PCOS OR Polycystic Ovary Syndrome) AND (adolescence* OR girl*) yielding 139 records. The obtained records were reviewed on the basis of titles and abstracts by two independent researchers. Full texts were reviewed and after rejection of systematic reviews, case reports and guidelines, 26 articles were selected for this study. Differences in search results between authors were resolved through discussion. A detailed diagram of the search strategy is presented in **Figure 1**.

## 3 Results

### 3.1 Treatment

#### 3.1.1 Non-pharmacological treatment

In the treatment of PCOS in teenage patients, the importance of non-pharmacological treatment and the need for lifestyle modification are increasingly emphasized. A study conducted during the COVID-19 pandemic in Iranian schoolgirls investigated the effect of 12 weeks of aerobic exercise on the hormone levels and lipid profile of girls with polycystic ovary syndrome (PCOS). The results of the analysis showed that 12-week aerobic exercise statistically significantly decreased testosterone, prolactin, estrogen, body weight, BMI, cholesterol, triglycerides and low-density lipoprotein levels, while high-density lipoprotein levels increased. The above results indicate that aerobic exercise has a positive effect on the health of girls with PCOS and is an effective and non-invasive method that should be an integral part of therapy (13).

#### 3.1.2 Pharmacological treatment

**Figure 2:**
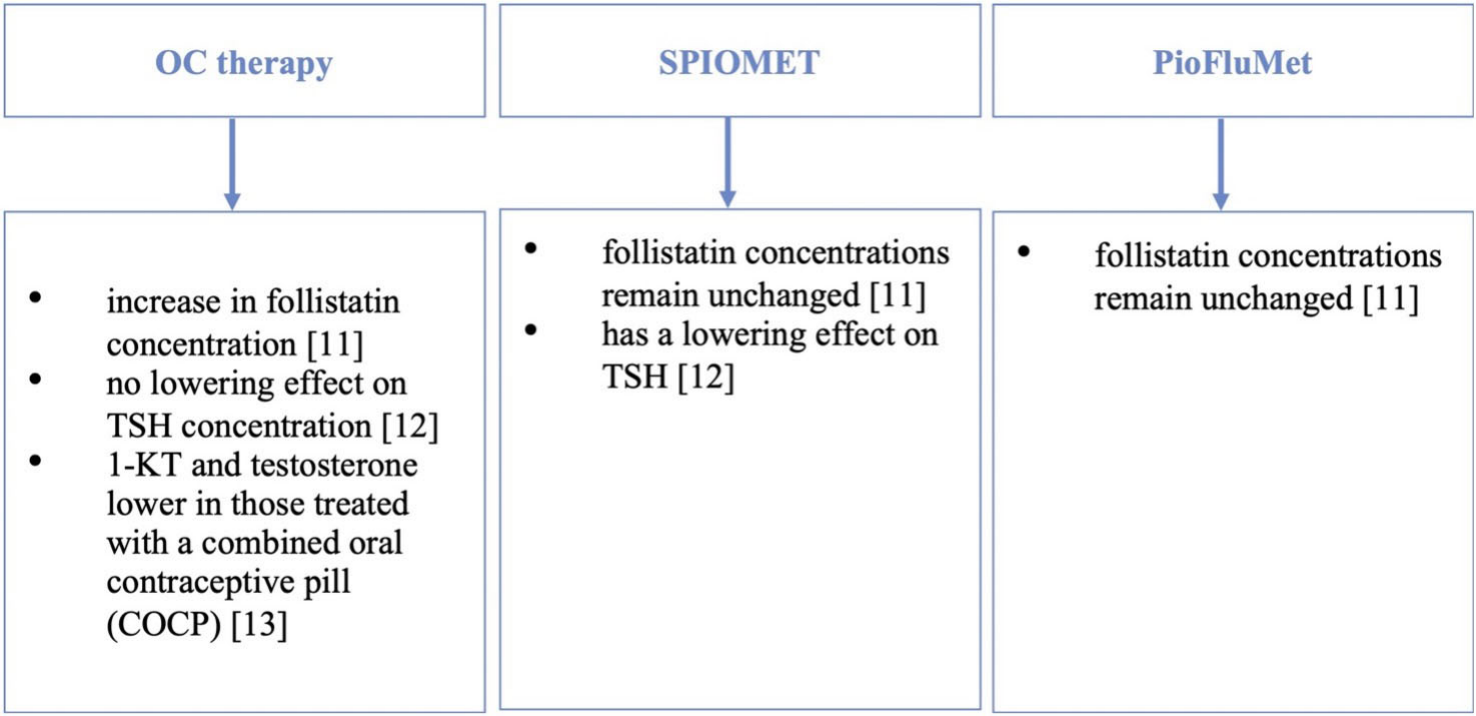
Treatment of PCOS in adolescent girls - information summary.

The effect of therapy with oral contraceptives (OC), the combination of spironolactone-pioglitazone-metformin (SPIOMET), the combination of pioglitazone-metformin-flutamide (PioFluMet) is also widely studied. Following reports that higher levels of follistatin are associated with an increased risk of type 2 diabetes and PCOS, a paper presented in Frontiers in Endocrinology investigated the effects of treatment of non-obese adolescent girls with PCOS on follistatin concentrations. Follistatin concentrations have been shown to increase with OC therapy but remain unchanged with the combination of PioFluMet or SPIOMET. Follistatin levels increase significantly after 6 months of OC use. Moreover, in the studied population of girls with PCOS, follistatin positively correlated with the average serum insulin concentration during the OGTT and with changes in the fat content in the liver (14). Another study examined the concentration of thyroid stimulating hormone (TSH) in adolescent PCOS patients and the effect of OC treatment and low-dose SPIOMET on TSH. It was shown that the mean TSH values were higher in PCOS patients than in girls from the control group. In addition, TSH levels varied between OC and SPIOMET treatment, remaining elevated with OC and falling rapidly with SPIOMET. Post-treatment TSH levels were stable in both subgroups. During treatment, TSH levels were not associated with changes in circulating androgens or total body weight but were consistent with changes in hepatic fat (15). The effect of PCOS treatment on androgen levels has also been studied. 11-ketotestosterone (11-KT) and testosterone were lower in those treated with a combined oral contraceptive pill (COCP) compared to untreated PCOS. With metformin treatment, testosterone levels were lower, but the treatment had no effect on 11-oxyandrogens (16). A summary of treatment information is shown in **Figure 2**.

One of the widely studied issues regarding the pathophysiology of PCOS is the mismatch sequence. This sequence is characterized by less weight gain in the prenatal period and more in the postnatal period. Consequently, it can lead to ectopic lipid accumulation, trigger the development of early adrenarche/pubarche and activate the gonadotropic axis leading to early puberty and development of PCOS. An ongoing randomized, placebo-controlled, multicenter study investigates whether appropriate pharmacological intervention can positively influence patients presenting with a mismatch sequence. The half-dose version of SPIOMET (mini-spiomet) is used in the study group. During this study, patients are monitored for parameters such as i.a. bone age or hormones concentration. The study is to answer whether thanks to pharmacological intervention there is a chance to slow down too fast puberty and achieve a reduction of ectopic adipose tissue without the need to reduce body weight in girls with a mismatch sequence. For conclusions we have to wait till 2026 (17).

Due to the fact that taking metformin (MET) may reduce the absorption of vitamin B12, a study was conducted to verify whether there are deficiencies of this vitamin in a group of teenage patients during MET therapy. In a case control study, no differences in vitamin B12 concentration were found between children and adolescents undergoing MET therapy compared to the control group. In children and adolescents undergoing metformin therapy (MET), the intake of vitamin B12 in the diet is suboptimal, which in combination with MET may lead to vitamin B12 deficiency. Therefore, caution should be exercised when prescribing MET to children and adolescents, and attention should be paid to reliable education of the patient and the patient’s parents on the principles of rational nutrition (18).

Due to the high prevalence of depressive disorders among PCOS patients, a study in Histochemistry and Cell Biology presented the results of a rat study that examined the effects of amitriptyline, a drug used to treat depression, on PCOS symptoms. In laboratory studies, serum FSH concentrations and the volume of the corpus luteum were shown to decrease in the PCOS + Ami group (amitriptyline-treated rats) compared to the PCOS group (amitriptyline-no-administered rats); however, amitriptyline did not affect the morphological and biochemical changes caused by PCOS in the ovarian tissues and degenerative areas. Taking into account all the results, it was concluded that Ami does not have a protective or healing effect on ovarian follicles and hormone levels, although it may have antioxidant activity capable of reducing oxidative stress in the presence of PCOS (19).

Other substances that can support the treatment of PCOS in adolescents are also constantly being sought. In a study conducted on a group of both teenage and adult patients (aged 15-34), it was shown that taking alpha-lipoic acid (800 mg/day) for six months resulted in an increase in the number of menstrual cycles during the 6-month study period and a decrease in BMI in the population with normoinsulinemia. However, more detailed research on the use of this substance in supporting treatment is necessary (20). In another study, the relationship between the level of vitamin D, leptin, AMH, HOMA-IR and FGF23 was checked, and no statistically significant correlation was found between the tested parameters and the concentration of vitamin D. Based on the results, it was concluded that additional vitamin D supplementation would not reduce the symptoms of polycystic ovary syndrome (21).

The study, which is a qualitative content analysis, showed that the necessary educational and advisory activities should be undertaken in the field of healthy eating and support for adolescent girls with PCOS. It was also emphasized that it is important to develop appropriate care structures for these patients and to solve communication problems and build trust between members of the medical team and these girls to ensure the best quality of care (22).

**Figure 3:**
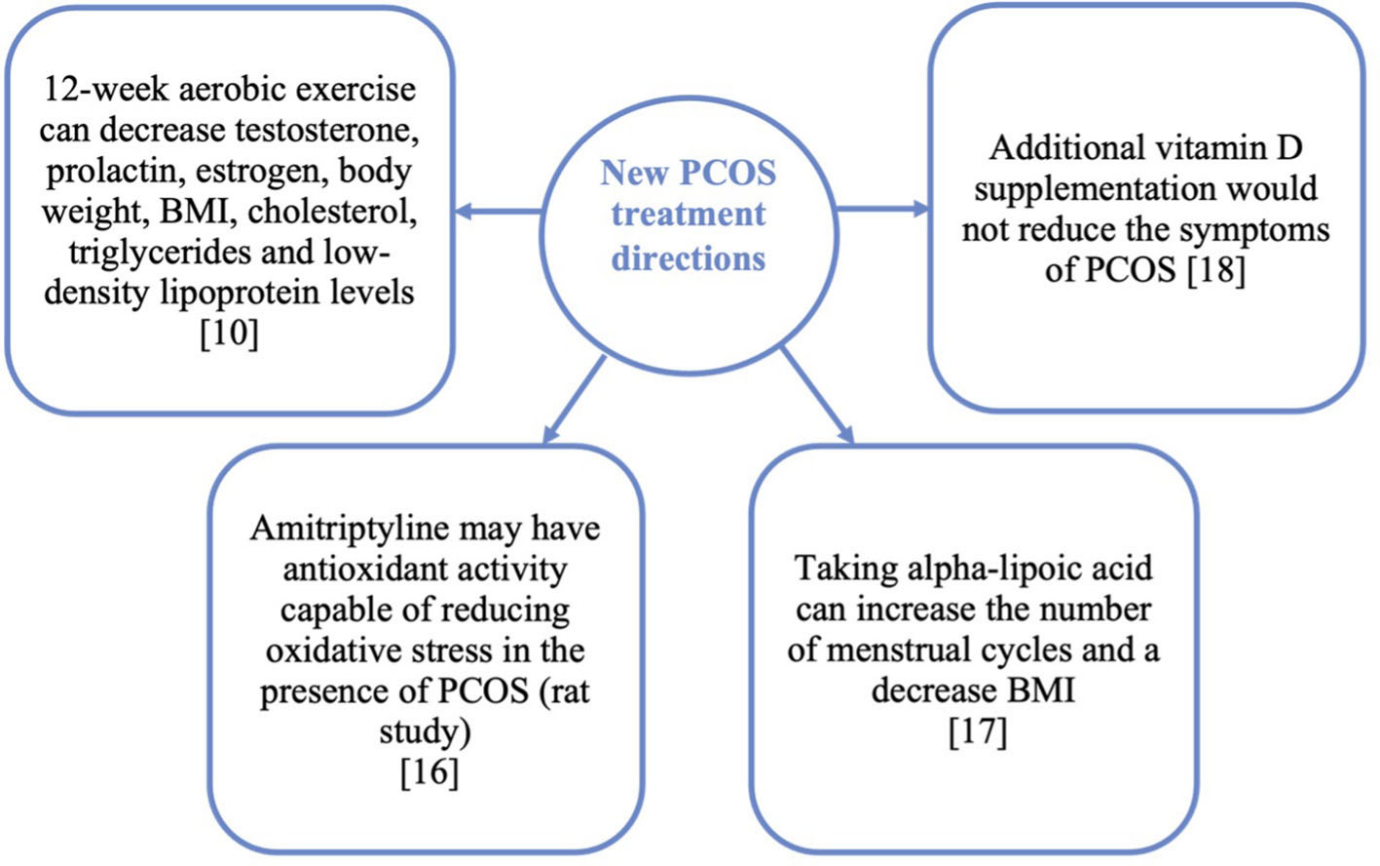
Summary of research topics conducted in the field of PCOS treatment in adolescent girls.

Summary of research in the subject of treatment is presented in **Figure 3**.

### 3.2 Complications and comorbidities

**Figure 4:**
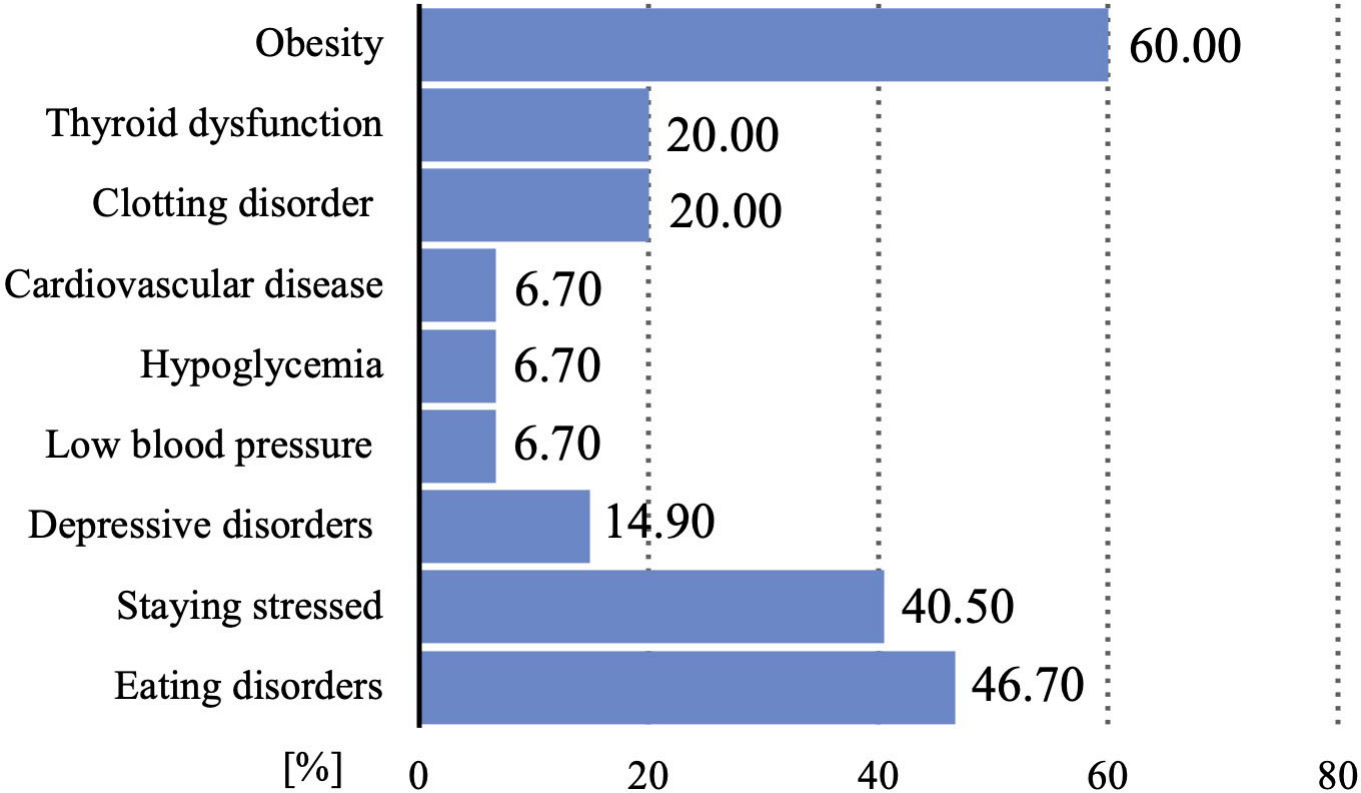
Prevalence of disorders among adolescents with PCOS or irregular menstrual cycles [%].

**Figure 5:**
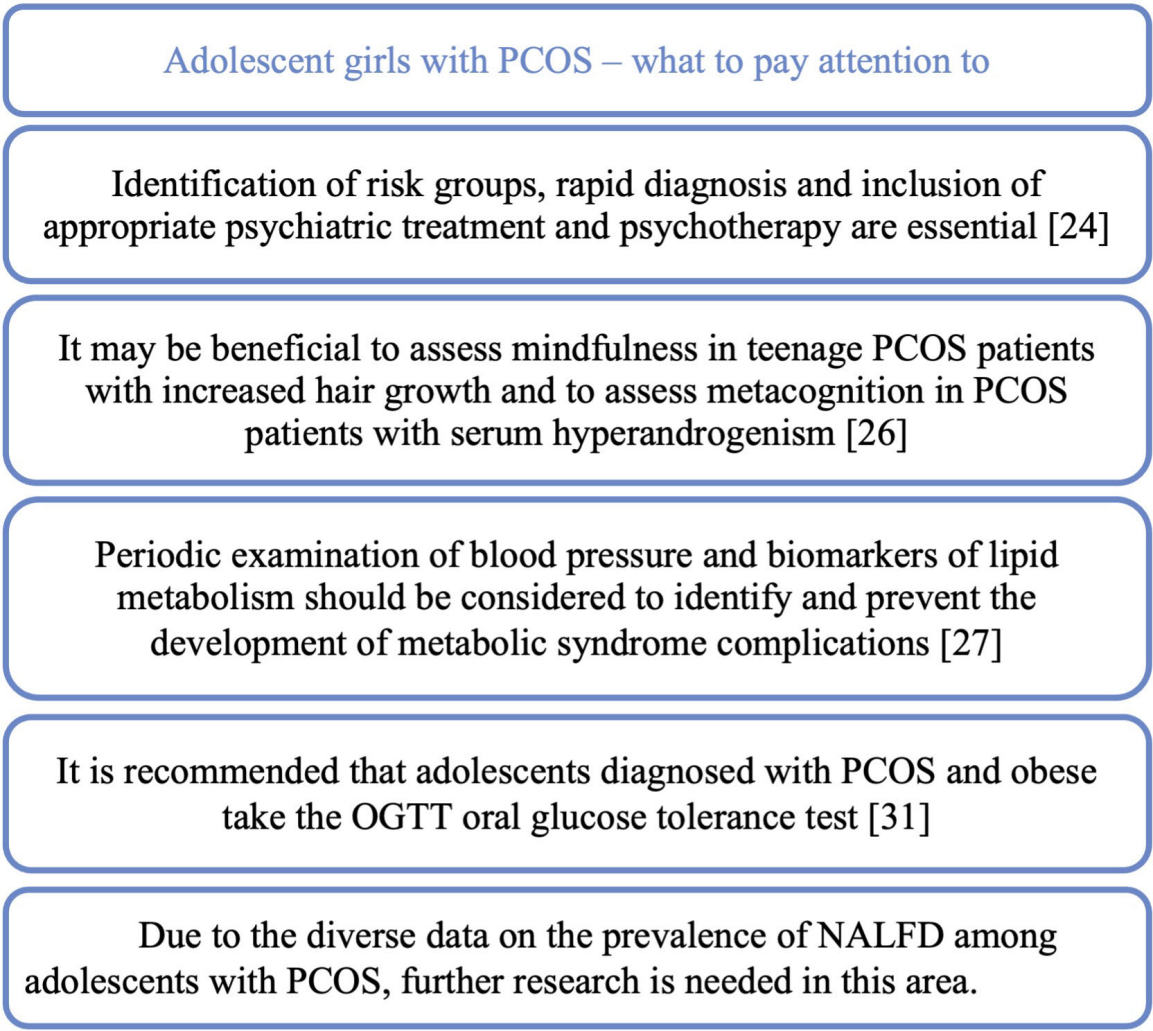
A teenage patient with PCOS – what should we pay attention to in the context of comorbidities and possible complications.

As a consequence, PCOS may lead to many complications and the coexistence of other diseases, including: endocrine, cardiological, metabolic and mental. A study on adolescent population from India showed that among teenage PCOS patients comorbidities such as obesity (60%), thyroid dysfunction (20.0%), clotting disorder (20.0%), cardiovascular disease (6.7%), hypoglycemia (6.7%), low blood pressure (6.7%) (23) (**Figure 4**) Summary information on comorbidities and complications is provided in **Figure 5**.

#### 3.2.1 Mental health

The increasing incidence and severity of depressive disorders among the entire pediatric population, but also among teenage PCOS patients, is a major challenge for physicians treating this group of patients. The prevalence of depressive disorders among patients with irregular menstrual cycles was estimated at 14.9%, at the same time, as many as 40% of patients declare staying stressed. Eating disorders affect as many as 46.7% of patients in the study population (23). A meta-analysis showed that adolescents and young women with PCOS had significantly more depressive and anxiety symptoms than those without PCOS (24). Due to the importance of the problem of depressive disorders in teenage PCOS patients, a retrospective study was planned to construct and verify a depression warning. The aim is to examine what factors influence the risk of depression in adolescents with PCOS and how interpretable and accurate the LASSO-based (Least Absolute Shrinkage and Selection Operator) alert system is (25). An important issue in the management of teenage PCOS patients may be the correct way of communication. Therefore, a study has been planned to test the effectiveness of the psychological adjustment communication system in teenagers with PCOS who are at high risk of depression (26). Identification of risk groups, rapid diagnosis and inclusion of appropriate psychiatric treatment and psychotherapy are essential to improve the quality of life and prognosis of patients in terms of mental health. In study published in the Journal of Pediatric Nursing teenagers with PCOS were shown to have significantly lower the body image concerns inventory (BICI) total scores and were more likely to have body image concerns. Hyperandrogenism or obesity alone, without the presence of PCOS, had no statistically significant negative effect on the BICI total score. Moreover, the study showed that, independently of PCOS, abnormal uterine bleeding (AUB) was associated with significant changes in the BICI and its domains (27). Another study constructed and validated a health-related quality of life (HRQOL) 20-item questionnaire (APQ-20) for adolescent PCOS covering topics such as emotions and mood, loss of attractiveness due to hirsutism, loss of attractiveness due to acne, self-care, support and menstrual cycle problem. Using this questionnaire could be a quick way to identify patients with poor quality of life and suggest appropriate steps to improve the patient’s situation (28). Researchers also became interested in the effect of hyperandrogenism and obesity on mindfulness and metacognition. The assessment was based on the Mindful Attention Awareness Scale (MAAS) and the Metacognition Scale Child and Adolescent Form (MCQ-C). Mindfulness was shown to be lower in participants with a higher modified Ferriman-Gallwey score and in obese PCOS patients. In the group without hyperandrogenism, the result of Cognitive monitoring (MCQ-C-CM) was significantly higher, while in the group with hyperandrogenism, a positive correlation was detected between higher androgen levels and the positive meta-worry (MCQ-C-PM) sub-scale of the MCQ-C. In the MCQ-C-CM (Cognitive monitoring) and MCQ-C-PM (the positive meta-worry) subscales, the scores increased with increasing androgen levels. Therefore, it may be beneficial to assess mindfulness in teenage PCOS patients with increased hair growth and to assess metacognition in PCOS patients with serum hyperandrogenism (29).

#### 3.2.2 Metabolic health

The effect on metabolic disorders in PCOS patients is currently being extensively studied. In a meta-analysis, it was shown that teenagers with PCOS are more than three times more likely to develop the metabolic syndrome (MetS) than the control group. Among adolescents with PCOS and obesity, the incidence of MetS was higher and the concentration of triglycerides was higher than in obese patients without PCOS. Among adolescents with PCOS, systolic blood pressure was higher compared to these without PCOS. In view of the above results, the authors of the study suggest that periodic examination of blood pressure and biomarkers of lipid metabolism in adolescent PCOS patients should be considered in order to identify and prevent the development of metabolic syndrome complications in this group as soon as possible (30).

PCOS is associated with insulin resistance and may also coexist with type II diabetes. In the meta-analysis, which covered the population of patients aged 8.9 to 75 years with type 2 diabetes (T2DM), the incidence of PCOS in the group < 25 years was 18% (31). Fasting glucose and Insulin resistance (IR) markers HOMA-IR (Homeostasis model assessment of insulin resistance) and QUICKI (Quantitative insulin sensitivity check index) have been shown to be more altered in adolescents with PCOS compared to controls (32). Adolescents with PCOS were also shown to have significantly higher BMI, higher hip circumference (HC), lower high-density lipoprotein (HDLc) cholesterol, and higher levels of alanine aminotransferase (ALT) and gamma-glutamyltransferase (GGT) than the control group (32). Another study showed that an abnormal two-hour OGTT (Oral Glucose Tolerance Test) was found in one in five (21.6%) of teenage PCOS patients. Teenage patients with PCOS and obesity compared to the control group had significantly higher HOMA-IR and lower insulin sensitivity according to WBISI (Whole body insulin sensitivity index) (33).

Non-alcoholic fatty liver disease (NAFLD/MAFLD) is a common subject of research into the complications of PCOS. It was shown that teenagers with PCOS complicated by NAFLD accounted for 37.5% of the respondents, and those with coexisting obesity and lower SHBG were more predisposed to the development of NAFLD (34). In another study, the prevalence of NAFLD in adolescents with PCOS was found to be 19.1%, not significantly different from the prevalence in adolescents without PCOS (16.8%). However, it was shown that patients with PCOS and concomitant type 2 diabetes (T2DM) had an increased risk of NAFLD (35). The incidence of NAFLD and the metabolic and hormonal risk factors associated with NAFLD in adolescents with PCOS was investigated also using non-invasive methods of diagnosis, VCTE elastography (Fibroscan) and ultrasonography (USG). The study found that the incidence of fatty liver features was similar in the PCOS and non-PCOS groups. However, when PCOS patients were categorized based on serum androgen levels, hyperandrogenic PCOS patients had a higher incidence of fatty liver than non-hyperandrogenic PCOS patients (36). In a prospective case–control study, the incidence of steatosis on ultrasound was 22.7% among adolescents with PCOS compared to 6.1% in the group without PCOS. Fatty liver index (FLI) was significantly higher in the PCOS group. LFS (liver fat score) indicating NAFLD occurred in 24.4% of the PCOS group, compared to 8.57% in the control group. NAFLD was diagnosed based on the hepatic steatosis index (HSI) in 40.9% of adolescents with PCOS, compared to 4.2% in the control group. In the liver fibrosis study with Fibroscan^®^, there was no significant difference between the PCOS and control groups. Adolescents with PCOS and a high free androgen index (FAI) presented worse NAFLD than adolescents with PCOS and a lower FAI (32). Due to the diverse data on the prevalence of NALFD among adolescents with PCOS, further research is needed in this area.

#### 3.2.3 Health monitoring

Markers that would allow for quick identification of patients at risk of developing metabolic complications are constantly sought. One of the potential markers studied is the level of telomerase, which, as shown by studies in the group of patients with PCOS as well as in the group of patients with the metabolic syndrome, was significantly lower than in the control groups (37). A study presented in Gynecological Endocrinology shows that the adipocyte factor visfatin has a certain clinical predictive value for IR and can be a protective factor in the development of metabolic syndrome in the course of PCOS. It was noted that visfatin will become a new target in the study of pathogenesis and early prevention of metabolic syndrome in teenage PCOS patients (38). HbA1c has been shown to be a poor test for impaired glucose tolerance (IGT) and it is recommended that adolescents diagnosed with PCOS and obese take the OGTT oral glucose tolerance test (33).

## 4 Discussion

In recent years, we have seen an increase in scientific interest in the subject of PCOS among the adolescent patient population and an increasing number of studies devoted to this issue. In our article, we have summarized the latest reports on the treatment of PCOS, comorbidities, and presented the current directions of research on PCOS in adolescents.

The analyzed studies emphasize that the inclusion of appropriate treatment and guiding the patient through appropriate lifestyle changes can significantly improve his quality of life. The literature review showed that it is necessary to individually select medications based on the patient’s current problems and to ensure that the treatment contributes to reducing the risk of developing other diseases that often occur in PCOS patients. The latest research on comorbidities with PCOS pays particular attention to two important aspects - the mental and metabolic health of patients. Due to the increasing prevalence of these diseases, also among teenage patients, it is important to diagnose and treat them as soon as possible to prevent future consequences.

It should be noted that despite the growing popularity of the topic of Polycystic Ovary Syndrome, most of the studies still concern the population of adult patients and further studies are necessary on the largest possible groups of teenage patients with PCOS to improve diagnostic and therapeutic algorithms.

The above review of current literature shows that a holistic approach and the creation of a multi-specialist patient care scheme may be of significant importance in the care of a patient with PCOS. The key seems to be the role of primary care physicians, including a pediatrician who takes care of a minor patient on a regular basis, in order to quickly and effectively detect risk factors and the possible need to deepen diagnostics towards PCOS. In addition to gynecological and endocrine care, it is necessary to ensure the care of Specialist Clinics, depending on comorbidities and complications - the care of Psychiatric, Cardiology and Diabetes Clinics seems to be of key importance. The possibility of psychological and dietary care, access to people creating an individual training plan who will take care of the patient in the field of lifestyle modification, which currently seems to be a key step in proper PCOS therapy, may be very helpful in managing a PCOS patient. Due to the growing problem of mental disorders among adolescents, in particular in patients with PCOS, special attention should be paid to the possibility of these disorders in patients and they should be immediately looked after by a psychiatrist and a psychologist. The introduction of screening tests for depressive disorders in this group of patients should be considered in order to detect the developing problem as soon as possible. Among the possible complications of PCOS, the occurrence of NAFLD in adolescents with PCOS is widely studied.

## Data Availability

The original contributions presented in the study are included in the article/supplementary material. Further inquiries can be directed to the corresponding author.
